# Baited-boats: an innovative way to control riverine tsetse, vectors of sleeping sickness in West Africa

**DOI:** 10.1186/s13071-015-0851-0

**Published:** 2015-04-18

**Authors:** Jean-Baptiste Rayaisse, Ernest Salou, Fabrice Courtin, Wilfrid Yoni, Issiaka Barry, Fabien Dofini, Moise Kagbadouno, Mamadou Camara, Stephen J Torr, Philippe Solano

**Affiliations:** Centre International de Recherche – Développement sur l’Elevage en zone Subhumide (CIRDES), Bobo-Dioulasso, Burkina Faso; Institut de Recherche pour le Développement, UMR 177 IRD-CIRAD INTERTRYP, CIRDES, Bobo-Dioulasso, Burkina Faso; Programme National de Lutte contre la Trypanosomiase Humaine (PNLTHA), Conakry, Guinea; Liverpool School of Tropical Medicine, Liverpool, UK; Warwick Medical School, University of Warwick, Coventry, UK; Institut de Recherche pour le Développement, UMR 177 IRD-CIRAD INTERTRYP, Montpellier, France

**Keywords:** Mangrove, Tsetse, Pirogue, Target

## Abstract

**Background:**

Human African Trypanosomiasis (HAT) is an important neglected tropical disease caused by *Trypanosoma* spp. parasites transmitted by species of tsetse fly (*Glossina* spp). The most important vectors of HAT are riverine tsetse and these can be controlled by attracting them to stationary baits such as insecticide-impregnated traps or targets deployed along the banks of rivers. However, the geographical nature of some riverine habitats, particularly mangroves but also extensive lake and river networks, makes deployment of baits difficult and limits their efficacy. It is known that tsetse are attracted by the movement of their hosts. Our hypothesis was that mounting a target on canoes typically used in Africa (‘pirogues’) would produce an effective means of attracting-and-killing riverine tsetse in extensive wetland habitats.

**Methods:**

In Folonzo, southern Burkina Faso, studies were made of the numbers of tsetse attracted to a target (75 × 50 cm) of blue cloth and netting mounted on a pirogue moving along a river, versus the same target placed on the riverbank. The targets were covered with a sticky film which caught tsetse as they contacted the target.

**Results:**

The pirogue-mounted target caught twice as many *G. tachinoides* and *G. p. gambiensis*, and 8 times more *G. morsitans submorsitans* than the stationary one (P < 0.001).

**Conclusion:**

Pirogues are common vehicle for navigating the rivers, lakes and swamps of West Africa. The demonstration that tsetse can be attracted to targets mounted on such boats suggests that pirogues might provide a cost-effective and convenient platform for deploying targets to control tsetse in the mangrove systems of West Africa where HAT persists. Further studies to assess the impact of pirogue-mounted targets on tsetse populations in HAT foci and the protective value of targets for pirogue passengers are recommended.

## Background

Tsetse flies transmit trypanosomes to humans and animals causing human and animal African trypanosomiases, commonly called sleeping sickness and nagana, respectively. Human African Trypanosomosis (HAT) is a deadly neglected tropical disease for which there is neither a vaccine nor effective chemopropylaxis, and none in prospect. Some drugs are available [1], but their use is still complicated and needs long hospitalisation.

The main important vectors of HAT in West Africa are riverine tsetse, mostly *Glossina fuscipes spp.* and *G. palpalis spp*., and the most intractable HAT foci are associated with mangrove habitats along the coastal regions of western and central Africa such as the Boffa and Dubreka foci in the Republic of Guinea [2,3], or mangrove foci in Equatorial Guinea and Gabon [3-5]. Access to these areas is particularly difficult and this hampers both detection and treatment of cases and vector control [2]. Despite prolonged medical efforts to control HAT, the disease often persists in these foci [2,3].

The inhabitants in these areas use canoe-like ‘pirogues’ to navigate through the swamps. Since most movements through the swamp are within a pirogue and tsetse are attracted to moving objects, it seems likely that this is also where most people are bitten. Indeed, studies of savannah tsetse suggest that passengers in a vehicle are particularly at risk of being bitten by infective tsetse [6-8]. Consequently there is a pressing need to develop novel methods to improve tsetse control, mainly in difficult access area. This improvement in tsetse control may additionally constitute a personal protection to people.

The main method of reducing the risk of being bitten by infective tsetse is through vector control. The most cost-effective method for HAT foci is the use of insecticidal baits: insecticide-treated livestock, and traps or targets made of insecticide–impregnated cloth which can be used to lure and kill tsetse. In mangrove swamps, livestock are scarce and hence the only feasible method is to deploy targets or traps [9-12]. These artificial baits are generally deployed on the banks of the rivers, in a stationary position, and kill the tsetse that contact them.

We propose using targets in a new way. Rather than only deploying targets evenly throughout a swamp, we propose that they should also be mounted on the pirogues used by local people. The targets will then be distributed to all areas where people live and work, and the attraction of tsetse to mobile objects may make the pirogue-mounted targets more effective than stationary ones. In this paper, we report the results of experiments designed to compare the numbers of tsetse attracted to mobile, pirogue-mounted targets driven by a human, versus the usual stationary targets. We show that many more tsetse are attracted to the “baited boats”, suggesting this may constitute an additional novel tool to control tsetse and HAT in mangrove habitats.

## Methods

### Study location and period

The experiment was undertaken in the Folonzo game reserve (~09° 54’ N, 04° 36’W), southern Burkina Faso, where no HAT occurs. In this area four tsetse species occur sympatrically [13] along the banks of the Comoe river. The riverine vegetation comprises a conserved gallery forest with *Syzygium guineense* as the predominant trees species [14]. The study was conducted in March – April 2013, during the hot-dry season when daily mean temperature and hygrometry in the gallery were 29 ± 2.7°C and 70 ± 14.9% respectively.

### Capture devices

**Figure 1:**
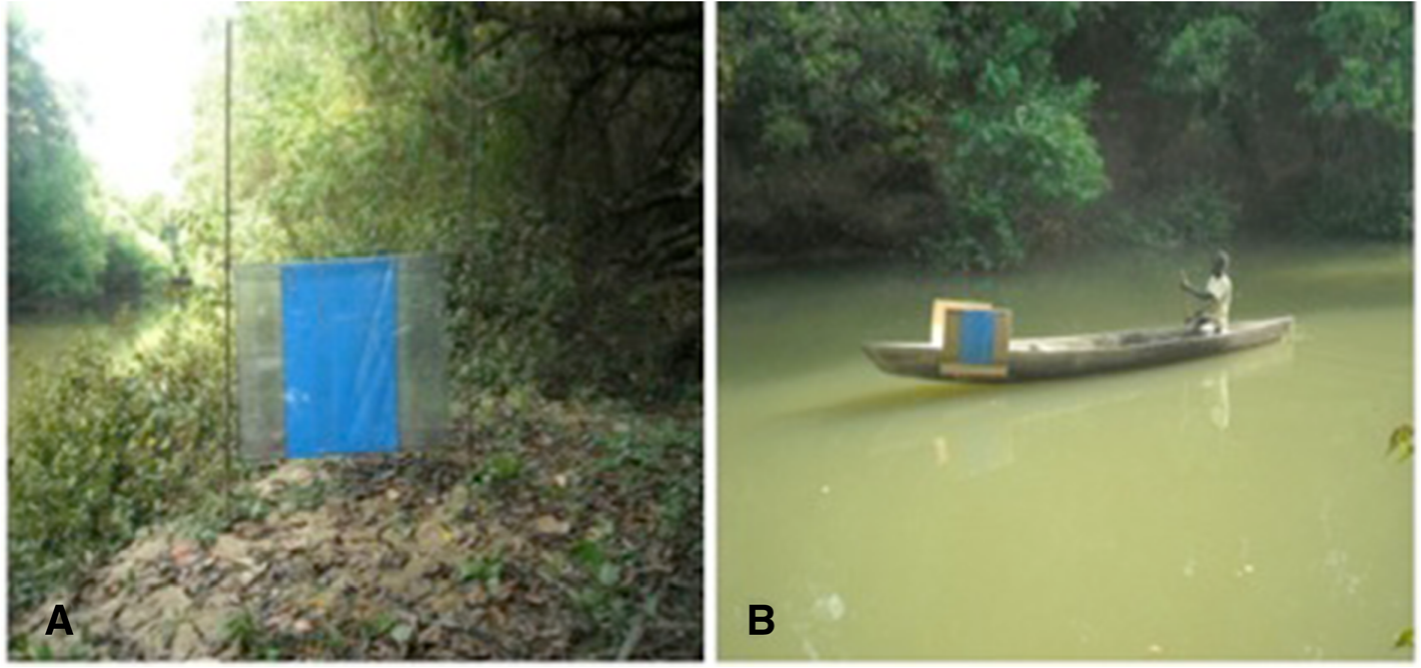
A stationary target on the bank of the comoé river **(A)** and a mobile “target on pirogue” **(B)**.

The study focussed on new designs of ‘tiny target’ which are currently being used in trials in Guinea and Burkina Faso [12]. The target has a central panel (0.5 × 37.5 m) of blue polyester flanked by two panels of black polyethylene netting (0.5 m high × 18.75 m wide each). To catch tsetse that contact the target, the cloth and netting panels were covered on the two faces with a sticky film (Luminos 4 film (1×20) – Ungridded; Renkotil Initial Supplies/UK), (see Figure 1A), [15-17]. The target was either operated at a single site on the riverbank, or mounted on a pirogue (Figure 1B) conducted by an individual using a paddle, which moved along the river at 1.1 m/s for ~100 m up and down the site during the two hours of capture. Hence, the target alone was compared to the whole system so called “target on pirogue”, constituted by the coxswain, the pirogue and the target.

The two systems were compared following a 2×2 Latin square design. In brief, when the pirogue was operated in the vicinity of Site 1 then the stationary target was operated on the bank at Site 2. The treatments were swapped between sites within each block of two days. The two sites were about 1.8 km from each other. Two independent experiments were conducted on this portion of the river, i.e. one in the morning (08:00 – 10:00 h) and the other one in the afternoon (15:00 – 17:00 h), with the comparison repeated 14 times for each period. For each given day (morning and afternoon), the treatment was kept on the same site and the transfer to another site was done according to a randomized rotation order.

### Statistical analysis

For every capture period (morning and afternoon), the species and sex of captured tsetse were recorded. For the comparisons of the two treatments, the catches were normalized and variances homogenized using a log_10_(n + 1) transformation, and then subjected to analysis of variance using Genstat (GenStat Discovery Edition 4) to assess whether the type of target (stationary or mobile) had a significant effect on tsetse catches.

To provide a common index of the effect of the target movements, the mean catch of tsetse from the target mounted on the pirogue is expressed as a proportion of that from the stationary one. The value is termed the catch index. Catch indices of 2 or 0.5 indicate that the target on pirogue caught twice or half as many tsetse as the stationary one, respectively. The results are presented as box plots produced using R [18] to show the median and quartiles.

## Results

Four species of tsetse (*Glossina tachinoides, G. palpalis gambiensis, G. morsitans submorsitans* and *G. medicorum*) were caught but due to the low number of *G. medicorum*, only the results for the first three species are presented.

A total of 6225 tsetse flies of the four species were captured during the two weeks trials (Table 1) comprising 66% *G. tachinoides*, 28% *G. p. gambiensis*, 5% *G. m. submorsitans* and 1% of *G. medicorum*. Catches of tsetse from both standard and mobile target were significantly greater in the afternoon. For *G. tachinoides*, 3× more males than females were caught in the afternoon on the stationary target, and 2.4× and 4.4× more males than females respectively in the morning and the afternoon for the pirogue-mounted target (P < 0.001).

**Table 1:** **Total number of flies caught per species, period and treatment during the 14 replicates of 2 hours trial in 14 days**

		***G. tachinoides***			***G. p. gambiensis***			***G. m. submorsitans***			***G. medicorum***			***All species***
**Treatment**	**Period**	**M.**	**F.**	**T.**	**M.**	**F.**	**T.**	**M.**	**F.**	**T.**	**M.**	**F.**	**T.**	
Stationary	Morning	313	239	552	127	72	199	27	16	43	2	1	3	797
	Afternoon	503	179	682	187	157	344	13	8	21	1	0	1	1048
Total stationary		816	418	1234	314	229	543	40	24	64	3	1	4	1845
Pirogue	Morning	840	346	1186	477	286	763	23	26	49	22	16	38	2036
	Afternoon	1387	312	1699	259	206	465	81	99	180	0	0	0	2344
Total pirogue		2227	658	2885	736	492	1228	104	125	229	22	16	38	4380
Global total		3043	1076	4119	1050	721	1771	144	149	293	25	17	42	6225
Global total (%)				66			28			5			1	100

M = males, F = females, T = total.

For the other two species, males also predominated but not significantly for both trapping devices.

### Comparison of trapping devices

#### Captures of G. p. gambiensis

**Figure 2:**
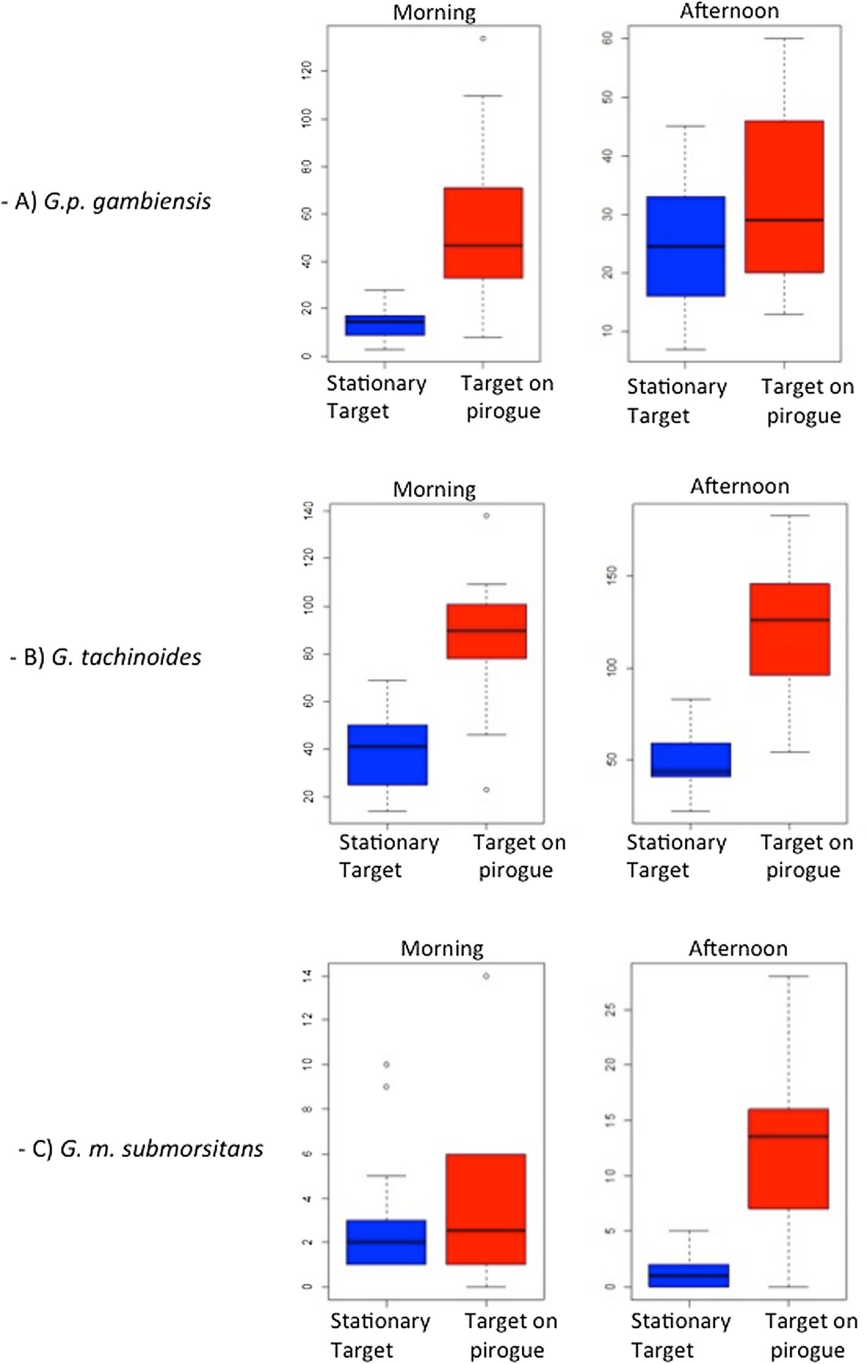
Median catches of *G.p. gambiensis* **(A)**, *G. tachinoides* **(B)** and *G. m. submorsitans* **(C)** following captures device and period. The limits of the boxes indicate the twenty-fifth and seventy-fifth percentiles; the solid line in the box is the median; the capped bars indicate the tenth and the ninetieth percentiles, and data points outside these limits are plotted as circles.

The pirogue-mounted target, the human included, caught significantly more *G. p. gambiensis* than the stationary one. In the morning, the median catch of both sexes were 14.4 (interquartile range (IQR) 9.47 - 16.74) for the stationary target compared to 46.3 (IQR 33.73 - 67.79) for the one on the pirogue (Figure 2A). The difference between the two types of devices was highly significant (P < 0.001), with a catch index of 3.61 for the target on pirogue. Although the catch index was lower in the afternoon (1.39), the difference was still highly significant (P < 0.001) in favour of the target on pirogue. The median for the stationary target was 24.5 (IQR16 - 31.95) and the one for the target on pirogue was 29.0 (IQR 20.48 - 44.97) (Figure 2A).

#### Captures of G. tachinoides

As for *G. p. gambiensis*, the pirogue-mounted target caught more *G. tachinoides* than the stationary one. The catch indexes were similar for the different periods of the day (2.8 in the morning and 2.5 in the afternoon), and the difference between the two types of devices was significant (P < 0.001 for each period). The median in the morning was 41 flies/target (IQR 27.01- 48.43) for the stationary target and 90.0(IQR 78.50 - 99.47) for the target on pirogue. In the afternoon, these values were 44.0 (IQR 41.25 – 58.24) for the stationary target and 126.5 (IQR 98.18 – 145.50) for the one on the pirogue (Figure 2B).

#### Captures of G. m. submorsitans

There was no difference between the two devices in the morning which gave a catch index almost equal to 1. The median was 2 flies (IQR 1 – 3) for the stationary target and 2.46 (IQR 1 – 5.74) for the one on pirogue. In the afternoon, the medians were 1.0 (IQR 0.19 – 2) for the stationary target and 13.4 (IQR 7.24 – 16) for the target on the pirogue. The mean catch index was higher (8.77) for the target on pirogue with the human, p < 0.001.

## Discussion

This study assessed the performance of an innovative way of using a tool to kill tsetse, which has the prospects of protecting humans against tsetse in wetland habitats where other control methods are not effective enough. This tool, called a “baited boat”, consists of an insecticide-impregnated piece of blue cloth and net that is mounted on a pirogue and that attracts and kill tsetse. Our results show that this mobile target attracts and kills many more tsetse than the stationary one. This offers the exciting prospect of a new method to control tsetse in some of the most difficult HAT foci: mangrove habitats where sleeping sickness persists and is difficult to control [2,3].

### Tsetse flies presence and composition

In this work, we confirmed both the presence of the four tsetse species in the area, as previously reported [10,11], as well as the predominance of *Gt* over the other tsetse species. Here it has to be noted that the attractive devices were set either on the water or in the gallery on the bank of the rivers, habitat of the riverine tsetse species *Gt* and *Gpg. Gms*, which belongs to the so-called “savannah” species, is present at high densities in the area, but it occurs more in the savannah which explains why it was caught in relatively low numbers in the gallery.

The general trend is that more tsetse were caught in the afternoon than in the morning, particularly for *Gms* for which catches were 4× greater in the afternoon than the morning. Previous studies also found that evening catches were higher than morning catches for *G.m. morsitans* and *G. pallidipes* in Zimbabwe, and the differences were greater for stationary than for mobile baits [6].

### Comparison between systems

Whatever the species and the time of day, the pirogue-mounted target with the human always caught more tsetse than the stationary one. It is the first time that such a result is obtained for riverine species *G. tachinoides* and *G. p. gambiensis*. As a tentative of explanation, it is already known that species of the morsitans group are highly responsive to mobile baits. This has led to the development of mobile devices to attract and kill these tsetse on the ground [19,20]. In the laboratory, Brady demonstrated an activation response to moving targets that was correlated with time since feeding [8]. The same demonstration was also done in Zimbabwe where tsetse (*G. pallidipes* and *G. m. morsitans*, both from the morsitans group) were attracted to mobile visual baits, with greater response for hungry tsetse [7,21]. Presumably tsetse are activated by the visual stimulus of a host passing by. In addition, moving the target by 100 m around a fixed position increases its range of action, which is limited to 50 – 100 m in dense vegetation like the one in the Folonzo area, what may partially explain the results. The use of this tool in a new way may also be a powerful tool for monitoring populations of riverine tsetse, such as mobile baits that are used to monitor subspecies of *G. morsitans*.

Vale [7], suggested that mobile baits mainly recruit resting tsetse whereas stationary baits recruit ranging flies. The differing compositions of catches from the two types of bait are then explained most simply by the hypothesis that responsiveness in the resting condition is greater for males than for females and began early in the hunger cycle, whereas ranging begins later and does not differ greatly according to sex and species. Hargrove [6], also said that a large part of the response of males is because they are seeking females. Further studies are necessary for a better understanding of the role of sex and age in the tsetse behaviour.

## Conclusion

As a conclusion, a target mounted on a pirogue (“baited boat”) significantly caught more tsetse than a stationary one. This device seems a promising tool to suppress tsetse populations and serve to protect humans in habitats where tsetse control is difficult to implement, i.e. mangrove and rivers, in the absence of any chemoprohylaxis available. In these habitats, the pirogue is the most common mean of work and displacement, and this tool seems adapted to that. Further work should assess the persistence and performance of insecticide on targets in these humid settings, and the acceptability of this control method to local communities (fishers, and people using the pirogues).

### Ethical approval

Our experiment is in conformity with our institution ethical rules and the only one human (the coxswain) who involved in the trial was adult and consenting. As a matter of fact he is a native of the locality and used to do the same activity under the same conditions.
